# Aberrant Signaling Pathways in T-Cell Acute Lymphoblastic Leukemia

**DOI:** 10.3390/ijms18091904

**Published:** 2017-09-05

**Authors:** Deborah Bongiovanni, Valentina Saccomani, Erich Piovan

**Affiliations:** 1 Dipartimento di Scienze Chirurgiche, Oncologiche e Gastroenterologiche, Universita’ di Padova, Padova 35128, Italy; deborah.bongiovanni@studenti.unipd.it(D.B.); valentina.saccomani@studenti.unipd.it (V.S.); 2 UOC Immunologia e Diagnostica Molecolare Oncologica, Istituto Oncologico Veneto IOV—IRCCS, Padova 35128, Italy

**Keywords:** acute lymphoblastic leukemia, oncogenes, PI3K/AKT, targeted therapy

## Abstract

T-cell acute lymphoblastic leukemia (T-ALL) is an aggressive disease caused by the malignant transformation of immature progenitors primed towards T-cell development. Clinically, T-ALL patients present with diffuse infiltration of the bone marrow by immature T-cell blasts high blood cell counts, mediastinal involvement, and diffusion to the central nervous system. In the past decade, the genomic landscape of T-ALL has been the target of intense research. The identification of specific genomic alterations has contributed to identify strong oncogenic drivers and signaling pathways regulating leukemia growth. Notwithstanding, T-ALL patients are still treated with high-dose multiagent chemotherapy, potentially exposing these patients to considerable acute and long-term side effects. This review summarizes recent advances in our understanding of the signaling pathways relevant for the pathogenesis of T-ALL and the opportunities offered for targeted therapy.

## 1. Introduction

T-cell acute lymphoblastic leukemia (T-ALL) is an aggressive hematological tumor arising from the malignant transformation and subsequent clonal expansion of T-cell precursors expressing immature T-cell markers. Based on the maturational stage of normal thymic development at which transformation occurs, T-ALL can be classically sub-classified into early cortical, late cortical or mature [1]. Recently, a new subgroup called Early T-lineage progenitor T-ALL (ETP-ALL) which shows a block at the earliest stages of T-cell differentiation and lacks expression of several T-cell surface markers [Cluster of Differentiation (CD)1a, CD8, and CD5] but instead expresses myeloid and stem cell markers, has been described [2,3]. T-ALL accounts for 25% of adult and 15% of pediatric ALL cases, with cure rates respectively reaching 60% and over 80% of the patients thanks to current intensive chemotherapy protocols [4]. However, primary resistance to treatment and relapse are observed in a significant number of patients, for which prognosis remains poor, underscoring the need of more efficient and specific treatments.

At the molecular level T-ALL is a heterogeneous disease, where a wide spectrum of genetic lesions [5] and microenvironmental factors [6] cooperate to promote a multistep leukemogenic process, altering normal cell growth, survival, proliferation, and differentiation during thymocyte development. Notwithstanding, genetic alterations that activate neurogenic locus notch homolog protein 1 (NOTCH1) signaling and T-cell transcription factors, coupled with inactivation of the inhibitors of CDK4/alternate reading frame (INK4/ARF) tumor suppressors, are hallmarks of T-ALL [5,7,8]. Despite the prominent role of cell-intrinsic mechanisms in T-cell transformation, T-ALL cell growth is not fully cell autonomous and it is becoming clear that the metabolic milieu of the microenvironment dictates the behavior of tumors.

In this review, we summarize and discuss recent advances in understanding the signal transduction pathways that are de-regulated in T-ALL, the emerging role in cancer development of leukemic adaptation to metabolic stresses encountered in the native environment and evaluate their potential as therapeutic targets.

## 2. Normal T Lymphocyte Development

Differently from other hematopoietic lineages, development of T-cells from pluripotent hematopoietic stem cells takes place in the thymus. Early T-cell progenitors enter the thymus at the cortico-medullary junction [9] where they encounter instructive signals inducing cell growth and proliferation mainly through stimuli such as interleukin-7 (IL-7) [10,11] and stem cell factor (SCF) [12]. These uncommitted lymphoid progenitors activate NOTCH1 signaling while circulating along the thymic cortex, thus receiving instructive signals leading to cell lineage commitment toward a T-cell fate [13]. Several developmental stages of thymocyte differentiation can be distinguished through cell surface markers [14]. Thymocytes are primarily subdivided into double negative (DN), double positive (DP) and single positive (SP) subsets in relation to the expression of the molecules CD4 and CD8. The most immature thymocytes (early uncommitted thymocytes), named DN, can be subdivided into four stages of differentiation based on the expression of CD44 and CD25 (recently reviewed in Shah and Zúniga-Pflücker [15]). During the DN3 (CD44^−^CD25^+^) stage of development, the expression of a constitutively active pre-T cell receptor (pre-TCR; composed of the invariable pre-T alpha chain and the rearranged TCRβ chain) [16], induces very high levels of NOTCH1 signaling leading to marked cell proliferation. DN3 thymocytes then progress to DN4 cells (CD44^−^CD25^−^) and then intermediate single-positive (ISP) cells, a transition population that expresses a co-receptor (CD4 in humans or CD8 in mice) in the absence of high levels of surface CD3. NOTCH1 activation in these rapidly proliferating pre-TCR expressing T-cell committed progenitors in combination with Phosphatidylinositol 3-kinase (PI3K) signaling [17] is critical for supporting their maturation to CD4 ^+^ CD8^+^ DP cells. T-cell commitment towards the T-cell receptor alpha/beta (TCRαβ) positive lineage takes place during DN stages of development at which gamma delta (γδ) T-cells split off. When thymocyte precursors differentiate to DP cells, they cease to proliferate and rearrange their TCRα loci. These cells represent about 85% of all thymocytes and most express CD1a. After undergoing positive checkpoint selection (capacity of DP T-cells to recognize self-major histocompatibility complex (MHC) molecules) and negative checkpoint selection (capacity to recognize self-peptide loaded MHCs without eliciting an autoimmune-prone response), thymocytes that express functional TCRs commit to either CD4 or CD8 SP lineage. SP CD4^+^ and CD8^+^ cells that survive negative selection leave the thymus and populate the peripheral lymphoid tissues.

## 3. Genetics of T-ALL

Among the genetic abnormalities, chromosomal translocations of transcription factor genes to regulatory regions of T-cell receptor genes are frequent and characteristic events of T-ALL. These transcription factors include basic helix-loop-helix (bHLH) family members such as T-cell acute lymphobastic leukemia 1 (TAL1) [18], TAL2 [19], lymphoblastic leukaemia associated haematopoietic regulator 1 (*LYL1*) [20], and Basic Domain, Helix-Loop-Helix Protein, Class B, 1 (*BHLHBL1*) [also known as Oligodendrocyte Transcription Factor 2 (*OLIG2*)] [21]; LIM-only domain (LMO) genes (*LMO1* and *LMO2*) [22,23]; homeobox (HOX) transcription factors such as T-cell leukemia homeobox 1 (*TLX1/HOX11*) [24], *TLX3* (*HOX11L2*) [25], NK2 homeobox *NKX2-1*, *NKX2-2* and *NKX2-5* [26,27], and homeobox A (*HOXA*) cluster [28]; and proto-oncogenes such as avian myelocytomatosis viral oncogene homolog *MYC* [29] and myeloblastosis transcriptional activator (*MYB*) [30,31]. In addition, mutations and deletions in tumor suppressor genes such as Wilms Tumor 1 (*WT1*), Lymphoid Enhancer Binding Factor 1 (*LEF1*), ETS Variant 6 (*ETV6*), B-Cell CLL/Lymphoma 11B (*BCL11B*), Runt Related Transcription Factor 1 (*RUNX1*) and GATA Binding Protein 3 (*GATA3*) contribute to the overall transcriptional deregulation of T-ALL, extensively reviewed in [5]. Activating mutations of *NOTCH1* are present in at least 60% of T-ALL [7], while rare additional rearrangements result in the expression of chimeric fusion genes involving Lysine Methyltransferase 2A (*KMT2A*)/Mixed Lineage Leukemia 1 (*MLL1*), *HOXA* genes, and tyrosine kinase genes such as Abelson murine leukemia viral oncogene homolog 1 (*ABL1*) [32,33]. Deletions involving the cyclin-dependent kinase inhibitor 2A/2B (*CDKN2A/CDKN2B*) cell cycle regulator loci are highly prevalent (>70% of T-ALL cases [8]); rarer chromosomal deletions recurrently inactivate retinoblastoma gene 1 (*RB1*) and cyclin-dependent kinase inhibitor 1B (*CDKN1B*) in some cases of T-ALL [31,34]. Several genes encoding epigenetic regulators and chromatin modifiers are also recurrently mutated in T-ALL, including Enhancer of zeste homolog 2 (*EZH2*), suppressor of zeste 12 homolog (*SUZ12*) and embryonic ectoderm development (*EED*) [35], which are members of the polycomb repressor complex 2 (PRC2); the plant homeodomain factor gene PHF6 [36]; the histone demethylase lysine demethylase 6A (*KDM6A*) [37]; and the deubiquitinating enzyme ubiquitin specific peptidase 7 (*USP7*) [38].

## 4. T-ALL Molecular Subgroups

All classification schemes of T-ALL are essentially based on a comparison with normal T-cell development [39]. Moreover, gene expression profiling studies have helped in the identification of T-ALL molecular subgroups that are characterized by specific transcriptional profiles and aberrant expression of specific T-ALL transcription factor oncogenes, often as a consequence of a chromosomal defect [1,40]. The largest subgroup is defined by ectopic TAL1 expression (in some cases together with LMO1/LMO2) characterized by a mature late cortical thymocyte immunophenotype (CD4^+^ CD8^+^ CD3^+^). Other major subgroups show mutually exclusive expression of TLX1, TLX3, HOXA9/10, LMO2, or NKX-2 [26,28]. Leukemias associated with TLX1, TLX3 or NKX-1,-2 activation have an early cortical immunophenotype (CD1a^+^ CD4^+^ CD8^+^). Furthermore, the ETP-ALL subgroup showing a block at the earliest stages of T-cell differentiation (CD4^−^ CD8^−^ cells) has been described and corresponds to immature T-ALLs expressing stem cell genes. This subgroup frequently shows aberrant expression of LMO2/LYL1 [1,41].

Recent sequencing studies have suggested that between 10 and 20 protein sequence altering mutations are present in T-ALL cells [42,43,44]. This sequential increase in mutational burden does not seem to occur randomly, as specific combinations of mutations are often found [41,45]. In fact, mutations affecting the PI3K/v-akt murine thymoma viral oncogene homolog (AKT) pathway (phosphatase and tensin homolog/*PTEN*, phosphoinositide-3-kinase regulatory subunit 1/*PIK3R1*) are highly prevalent in TAL1+ cases, suggesting a strong pressure for *PTEN* inactivation to occur in these cells, and that TAL1 over-expression cooperates with mutations affecting the PI3K/AKT pathway (especially *PTEN* inactivation) to promote T-cell transformation. Conversely, interleukin 7 receptor/Janus kinase/signal transducer and activator of transcription (IL-7R/JAK/STAT) signaling pathway or rat sarcoma viral oncogene homolog (*RAS*) mutations are frequently observed in immature T-ALL cases or TLX1/TLX3^+^ and HOXA^+^ cases. ETP-ALL shows genetically a lower prevalence of *NOTCH1* mutations and *CDKN2A* deletions, instead has a high prevalence of mutations in genes implicated in JAK/STAT signaling, Ras signaling and epigenetic regulation. Other interesting observations include, association between mutations in the putative epigenetic regulators PHD finger protein 6 (*PHF6*) [36], CCCTC-binding factor (*CTCF*) [41], *KMT2A* [41] and *WT1* [46] with the TLX3 rearrangement; mutations in the ubiquitin-specific protease *USP7* and TAL1^+^ cases; and mutations in protein phosphatase non-receptor type 2 (*PTPN2*) and TLX1^+^ cases [41].

## 5. Signaling Pathways Involved in the Development of T-ALL

**Figure 1:**
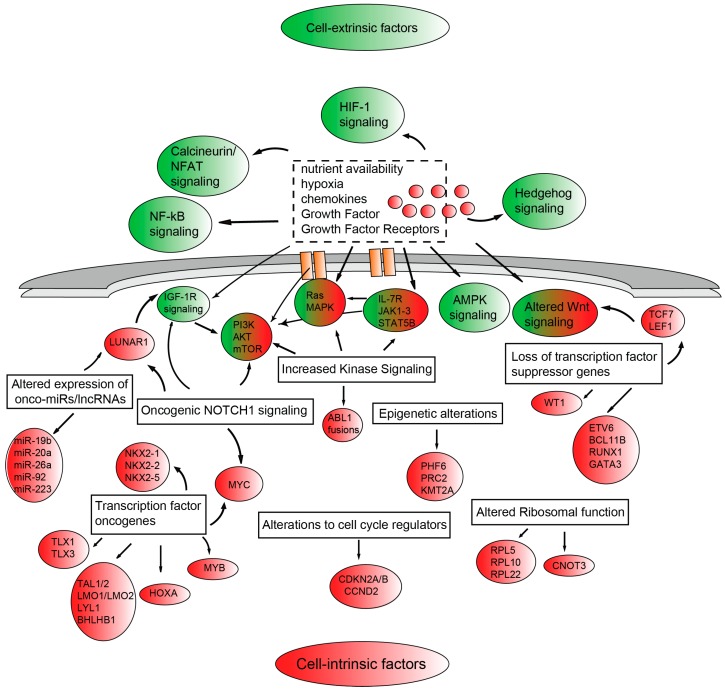
Schematic representation of signaling pathways aberrantly activated in T-cell acute lymphoblastic leukemia (T-ALL), tentatively subdivided as being mainly due to cell-extrinsic (shaded green) and cell-intrinsic factors (shaded red) or mixed (green and red).

The proliferative and survival advantages of T-ALL blasts result from altered signaling pathways which are frequently shared across the different molecular subgroups and crosstalk to promote disease progression (Figure 1). Frequently, the aberrant activation of these signaling pathways crucial to normal T-cell development and implicated in the pathogenesis of T-ALL, is due to genetic alterations of components of these pathways. However, it is becoming clear that bidirectional cross-talk between the microenvironment and leukemic cells exists and that microenvironmental cues ultimately contribute to tumor cell proliferation and survival [47]. Amongst the intracellular signaling pathways activated by genetic alterations (excluding oncogenic NOTCH1 signaling and *CDKN2A*/*CDKN2B* alterations) we find: (1) increased kinase signaling through: (i) the PI3K/AKT/mechanistic target of rapamycin (mTOR) [PI3K/AKT/mTOR] pathway, most commonly altered by *PTEN* deletion/mutations, *PTPN2* deletion, *PIK3R1* or v-akt murine thymoma viral oncogene homolog 1 (*AKT1*) mutations; (ii) the IL-7R/JAK/STAT pathway, via activating mutations in the interleukin 7 receptor α-chain gene (*IL-7Rα*), Janus kinase 1 (*JAK1*), *JAK3* or signal transducer and activator of transcription 5B (*STAT5B*); (iii) the RAS/mitogen-activated protein kinase (RAS/MAPK) signaling through Kirsten rat sarcoma viral oncogene homolog (*KRAS*) and neurofibromin 1 (*NF1*) mutations; and (iv) via chimeric ABL1 fusion genes such as nucleoporin 214kDa-Abelson murine leukemia viral oncogene homolog 1 (NUP214)-ABL1 and ETS Variant 6-Abelson murine leukemia viral oncogene homolog 1 (ETV6)-ABL1; (2) altered epigenetic regulation through mutations affecting (i) *PHF6*; (ii) PRC2 components *EZH2*, *SUZ12* and *EED*; (iii) *KMT2A* [also called ubiquitously transcribed tetratricopeptide repeat, X chromosome (*UTX*)] reviewed in [48]; (3) altered ribosomal function through mutations affecting Ribosomal protein L5 (*RPL5*), Ribosomal protein L10 (*RPL10*), Ribosomal protein L22 (*RPL22*) or CCR4-NOT transcription complex subunit 3 (*CNOT3*) [43]; and (4) altered expression of oncogenic miRNAs (onco-miRs) such as miR-19b, miR-20a, miR-26a, miR-92, and miR-223 or long noncoding RNAs (lncRNAs) such as leukemia-induced non coding activator RNA 1 (LUNAR1) [45]. On the other hand, cell-extrinsic microenvironmental factors such as nutrient availability, hypoxia, chemokines, growth factors and their receptors promote tumor growth through the activation of specific signal transduction pathways including PI3K/AKT/mTOR signaling, AMP-activated protein kinase (AMPK) signaling, Hedgehog signaling, calcineurin/nuclear factors of activated T-cells (NFAT) signaling, Wnt signaling and hypoxia-inducible factor (HIF-1) signaling.

### 5.1. Oncogenic NOTCH Signaling and NOTCH1-Myc Signaling Axis

The NOTCH1 signaling pathway is critical in the thymus for early T-cell fate specification and thymocyte development [49]. Aberrant activation of the NOTCH1 signaling cascade in T-ALL was first identified through the finding of a rare chromosomal translocation t(7; 9) (q34; q34.3) which determined the expression of a constitutively active form of NOTCH1 (Translocation associated notch protein 1; TAN-1) downstream of the TCRβ promoter [50]. However, this is a rare event and it took several years before NOTCH1 signaling entered the center stage of T-ALL biology. In fact, it was not until the year 2004, that activating mutations in *NOTCH1* were identified in over 60% of T-ALL cases [7]. A recent report using an integrated genomic approach in 264 T-ALL cases found an even higher frequency of *NOTCH1* mutations (≈75%) [41]. In addition, 8–30% of T-ALLs harbor mutations in F-box and beta-transducin (WD) repeat domain containing 7 (*FBXW7*), a protein that normally promotes NOTCH1 proteasomal degradation, and lead to increased NOTCH1 protein stability [51,52]. Moreover, paracrine mechanisms that result in NOTCH1 or neurogenic locus notch homolog protein 3 (NOTCH3) signaling upregulation or rare mutations in *NOTCH3* [53] may contribute to T-ALL. Thus, aberrant expression of the NOTCH ligand delta-like 4 (DLL4) may contribute to NOTCH1-driven leukemias [54]. The role of NOTCH1 signaling in the context of T-ALL and its intricate and complex interaction with c-MYC is discussed in detail in a recent review [55]. Here, we only discuss some of the main therapeutic implications of this signaling axis.

NOTCH promotes leukemia cell growth through direct transcriptional upregulation of anabolic pathways, including ribosome biosynthesis, protein translation and nucleotide and amino acid metabolism [56,57]. These growth-promoting effects of the NOTCH1 transcriptional program are enhanced by the upregulation of the MYC oncogene, a direct target of NOTCH1 [56,58,59]. Moreover, abrogation of oncogenic NOTCH1 signaling induces a metabolic crisis, which includes transcriptional down-regulation of anabolic genes, upregulation of catabolic pathways (ubiquitination, proteasome degradation), decreased glycolytic and glutaminolitic flux, and increased autophagy [58]. Interestingly, NOTCH1-dependent T-ALL cells are addicted to glutamine for cell growth, and genetic or pharmacological inhibition of glutaminase (enzyme that converts glutamine to glutamate for further processing in the Krebs cycle) has strong synergistic antitumor effects in combination with NOTCH1 inhibition.

The high prevalence of T-ALL cases having aberrant activation of the NOTCH-signaling pathway provides the rationale for the development of targeted therapies aimed at inhibiting NOTCH signaling in this disease [55]. Amongst the strategies adopted are: (i) the use of inhibitors of the proteolytic cleavage of the transmembrane NOTCH1 receptor by the presenilin/γ-secretase complex using γ-secretase inhibitors (GSIs), alone or in combination with vincristine or dexamethasone [60,61,62]; (ii) specific NOTCH1 inhibitory antibodies binding the negative regulatory region (NRR) of the NOTCH1 receptor [63,64]; (iii) stapled peptides such as SAHM1 that target the NOTCH1 transcriptional complex [65]; (iv) therapeutic targeting of downstream NOTCH pathway components such as the transcriptional target insulin-like growth factor receptor (IGF1R) [66] or (iv) inhibition of sarcoplasmic/endoplasmic reticulum calcium ATPase (SERCA) channels with thapsigargin which impairs the surface expression of mature NOTCH1 protein with preferential suppression of mutant NOTCH1 receptors [67]. Finally, hairy and enhancer of split 1 homolog (HES1) which plays an important role in T-cell development [68] and NOTCH1-induced leukemia, was recently confirmed as a critical downstream component of NOTCH1 signaling [69]. Interestingly, in this study perhexiline (a carnitine *O*-palmitoyltransferase 1 inhibitor, used in the treatment of angina) was able to evoke a strong anti-leukemic response in vitro and in vivo possibly by reverting the HES1 driven gene signature, providing a new lead for drug repurposing.

MYC is a master transcriptional factor that increases catabolism and is deregulated in numerous human cancers [70]. In early T-cell development, MYC controls cell growth downstream of NOTCH1 and pre-TCR signaling [71]. Although MYC has been shown to be a critical mediator of NOTCH1-dependent transformation [56,57,72], direct involvement of MYC overexpression in T-cell transformation was initially thought to occur only rarely due to translocations of MYC to the TCRα and TCRδ loci. Recently, however, the identification of massive binding of NOTCH1 at a distal enhancer near the MYC locus (NOTCH1-controlled MYC enhancer (N-Me)) has formally established a direct role for NOTCH1 in controlling MYC expression [58,59]. Moreover, recurrent somatically acquired focal duplications of the N-Me were found in 5% of T-ALL cases, providing an example of genetic alterations targeting oncogenic enhancer activity in T-ALL. MYC promotes cell transformation not only through increased cell growth and proliferation but also sustaining leukemia-initiating activity [73]. As mentioned above, MYC (and other oncogenic driver genes) may be aberrantly expressed through the intervention of super enhancers [74], broad areas of open chromatin characterized by high occupancy by bromodomain containing 4 (BRD4), p300, histone H3 lysine 27 (H3K27) acetylation, and the Mediator complex. Impacting on the activity of these large enhancers with molecules such as the BRD4 inhibitor S-Tert-butyl2-(4-(4-chlorophenyl)- 2,3,9-trimethyl-6H-thieno[3,2-f][1,2,4]triazolo[4,3-a][1,4]diazepin-6-yl)acetate(JQ1), may provide new opportunities for blocking oncogenic transcriptional networks [75].

### 5.2. PI3K/AKT/mTOR Pathway

The phosphoinositide 3-kinase (PI3K) pathway is one of the most frequently activated signal transduction cascades in human cancer. This family of lipid kinases plays a major regulatory role in several biological processes, including proliferation, survival, apoptosis, differentiation, migration and metabolism [76]. These numerous functions are served by distinct PI3K isoforms, which can be grouped into three classes according to their structure and substrate specificity. Class I PI3K, consisting of heterodimers of a catalytic subunit p110 and a regulatory subunit p85, is the most implicated in oncogenesis and is activated by growth factors through G protein-coupled receptors (GPCRs) and tyrosine kinase receptors (RTKs) [77]. PI3Ks phosphorylate phosphatidyl-inositol (4, 5) P2 (PIP2) to phosphatidyl-inositol (3, 4, 5) P3 (PIP3), a second messenger allowing the recruitment and activation of downstream effectors, such as phosphoinositide-dependent kinase 1 (PDK1) and AKT, via pleckstrin homology (PH) domain-mediated docking. The serine-threonine protein kinase AKT phosphorylates a wide range of cellular targets, including Mouse double minute 2 homolog (MDM2), Glycogen synthase kinase 3 beta (GSK-3β), Forkhead box protein O1 (FOXO1), Bcl-2-associated death promoter (Bad), Tuberous Sclerosis Complex 2 (TSC2)/tuberin and Caspase-9, and indirectly activates mechanistic target of rapamycin (mTOR) and nuclear factor kappa-light-chain-enhancer of activated B cells (NF-κB), generating an overall anti-apoptotic effect and proliferative signal [78,79]. Importantly, the phosphatase and tensin homolog (PTEN) tumor suppressor negatively regulates PI3K/AKT/mTOR signaling by dephosphorylating PIP3. In normal T-cells, PI3K/AKT cascade is a common effector pathway downstream of NOTCH, IL-7 and pre-TCR signaling, allowing their proper integration during early development and promoting survival of DN3 cells [80]. Indeed, different knock-out models resulting in defective signaling display a block in T-cell differentiation at the β-selection stage [81,82]. Moreover, the TCR and the co-stimulatory molecule CD28 signal through PI3K/AKT to promote survival, proliferation and cytokine production in activated T-cells [83,84].

In T-ALL, PI3K/AKT pathway is commonly constitutively activated [85,86], mainly as a result of the inactivation of PTEN, a lipid phosphatase functioning as the main negative regulator of PI3K pathway [85,87]. In primary T-ALL samples, *PTEN* deletions or loss-of-function mutations have been identified to cluster in exon 7, causing protein truncation at the carboxyl-terminus and its consequent degradation [85,86,88,89,90]. Less frequently, aberrant hyperactivation of the pathway due to gain-of-function mutations in PI3K regulatory (p85) and catalytic subunits (p110) (4, 5% of T-ALL cases) or in *AKT* (2, 3% cases) have also been reported [86]. However, genetic alterations are not sufficient to account for the very high frequency of PI3K signaling hyperactivation in T-ALL [87]. Indeed, non-genetic mechanisms, such as casein kinase 2 (CK2)-mediated phosphorylation and reactive oxygen species (ROS)-induced oxidation, can contribute to PTEN inactivation, affecting its lipid phosphatase activity [87]. Interestingly, normal and malignant thymocytes rapidly activate the PI3K/AKT/mTOR signaling pathway in response to IL-7 stimulation [91,92]. Thus, *IL-7R* activating mutations serve as an alternative mode of inducing enhanced PI3K/AKT/mTOR signaling in T-ALL cells. Moreover, NOTCH1 mediated transcriptional upregulation of IL-7Rα [93] and HES1-mediated transcriptional repression of PTEN [85] further contribute to enhance PI3K/AKT/mTOR signaling in NOTCH1 dependent T-ALL. A further level of complexity is added by the findings that PTEN mRNA can be targeted by miR-19 [94] or c-MYC [95] and that NOTCH1 likely regulates the dynamic exchanges of regulatory B subunits of protein phosphatase 2A (PP2A) leading to a decreased affinity of this phosphatase for critical targets such as phosphorylated AKT [96].

Moreover, insulin-like growth factors (IGFs) 1 and 2 bind to IGF1R, initiating a signaling cascade including PI3K/AKT and RAS/rapidly accelerated fibrosarcoma (RAF)/mitogen activated protein kinase (RAS/RAF/MAPK) (reviewed in [97]). IGF1R expression can be directly regulated by NOTCH1 and is important for T-ALL proliferation and leukemia initiating activity in vivo [66]. Additionally, IGF1R is a target of miR-223 and NOTCH1 can regulate IGF1R, at least in part by repressing miR-223 [98]. Moreover, long non-coding RNAs (lncRNA) such as LUNAR1 may contribute to NOTCH1 dependent IGF1R upregulation and signaling [99].

Given the widespread constitutive activation of PI3K/AKT/mTOR signaling, this signaling cascade has been explored as a novel therapeutic target in T-ALL. Experience with mTOR inhibition in T-ALL is limited, although recent data implicate mTOR activation in the development of early T progenitors and T-ALL [100]. In a mouse model of T-ALL evoked by Kras activation, Raptor deficiency, an essential component of mammalian target of rapamycin complex 1 (mTORC1), dramatically inhibited the cell cycle progression in oncogenic Kras-expressing T-cell progenitors, and specifically prevented the development of T-ALL [100]. On the other hand, mTORC1 inhibition by rapamycin prolonged survival of T-ALL bearing mice, but in the long run rapamycin-insensitive leukemia cells emerged leading to disease progression [100]. In addition, rapamycin may modulate glucocorticoid resistance, an important indicator of therapeutic failure in T-ALL [101]. However, through feedforward loops between mTOR, PI3K and AKT, inhibition of mTOR often leads to hyperactivation of AKT signaling [102]. This has spurred the search for other targets to hit therapeutically. At the level of PI3Ks, several pan-PI3K class I inhibitors (LY924002 and NVP-BKM120) showed anti-leukemic effects in T-ALL cell lines and primary patient samples having hyperactivation of PI3K/AKT pathway [103,104]. However, since only PI3Kδ and γ subunits are instrumental for T-cells, these constitute attractive targets to increase specificity and reduce toxicity. Elegant work demonstrated that the PI3Kδ and PI3Kγ isoforms are critically required to allow T-ALL development in a Pten-deficient mouse model [105]. In addition, the PI3Kδ/γ-specific inhibitor CAL-130 confirmed the “addiction” of Pten^null^ T-ALL on PI3Kδ and PI3Kγ by prolonging survival of Pten null mice. Similar efficacy was seen in PTEN-deficient primary human T-ALL samples [105]. Direct inhibition of AKT by the allosteric inhibitor MK-2206 has cytotoxic activity in some T-ALL cell lines and primary samples and may also target a putative leukemia-initiating cell (LIC) population [106]. Additionally, AKT inhibition sensitizes T-ALL cells to glucocorticoids [107]. To circumvent the possible issue of drug resistance seen with individual drug administration and problems related to feedforward loops, dual PI3K/mTOR inhibitors have been developed. In T-ALL cell lines and primary T-ALL cells, PI-103 showed promising cytotoxic effects [108,109]. Interestingly, the dual PI3K/mTOR inhibitor PI-103 determined upregulation of NOTCH1 target genes, including c-MYC. Accordingly, the combination of PI-103 with either a gamma secretase inhibitor (GSI) (such as L-685) or a c-MYC inhibitor (10058-F4) enhanced the effectiveness of PI-103. Given the overall convincing preclinical data on PI3K/AKT/mTOR signaling in T-ALL, but rather disappointing results of clinical trials [110], probably rational strategies of combination therapy will be needed.

### 5.3. IL-7R/JAK/STAT Pathway

Interleukin 7 (IL-7) is an essential cytokine for normal T-cell development and homeostasis that promotes cell survival and cell cycle progression. Upon ligand binding, the IL-7 receptor-α chain (IL-7Rα; CD127) and the common γ chain (γ_c_; CD132) dimerize and induce the transphosphorylation of JAK3 and JAK1. Activated JAKs phosphorylate the cytoplasmic tail of the receptor allowing the recruitment and phosphorylation of STAT5 (signal transducer and activator of transcription 5), which in turn dimerizes and translocates into the nucleus to regulate the transcription of target genes such as B-cell CLL/lymphoma2 (BCL-2) family members [111]. Besides JAK/STAT pathway, IL-7 can also mediate anti-apoptotic and proliferative signals via PI3K/AKT (Section 5.2) and RAS/MAPK pathways (Section 5.4). The expression of IL-7R is strictly regulated, as IL-7 is crucial at different stages of T-cell development for survival and maturation of specific cellular subsets in the thymus, as well as for the homeostasis of mature naïve and memory T-cells in the periphery (reviewed in [112,113]). Indeed, defective signaling in IL-7^−/−^ or IL-7R^−/−^ knock out mice results in early thymic development arrest and lymphopenia [114,115,116], while in humans inactivating mutations in the IL-7Rα [116] or γ_c_ [117] cause severe combined immunodeficiency.

Along with the pivotal role in T-cell biology, IL-7/IL-7R pathway has been also shown to promote leukemogenesis in vivo, since T- and B-cell lymphomas develop in IL-7 transgenic mice [118,119]. Analogously to normal T lymphocytes, T-ALL cells express IL-7R and stromal cells in the thymus and in the bone marrow—the microenvironments where T-ALL arises- secrete IL-7. Several studies have demonstrated that IL-7 can favor disease progression both in vitro and in vivo [120,121,122,123], regulating cell viability and proliferation. In T-ALL blasts, but not in healthy lymphocytes, IL-7 promotes cell cycle entry and cell viability, through the down-regulation of p27^kip1^ and up-regulation of BCL2 [91,124] in a PI3K/AKT-dependent manner [91,125]. Activating mutations in *IL-7Rα* have been identified in approximately 10% of T-ALL patients, most frequently leading to the introduction of a cysteine residue which allows ligand-independent homodimerization of the receptor and consequent constitutive signaling [126,127]. These mutations were found mainly within the TLX1/3, HOXA positive and ETP-ALL genetic subgroups [41,42]. Gain-of-function mutations in *JAK3* and *JAK1* [42,128,129], as well as in *STAT5* gene [130,131] have been detected in a variable fraction of T-ALL patients, along with a rare t(9;12)(p24;p13) translocation driving the expression of a constitutively active fusion protein ETV6-JAK2 [132,133]. It is also of relevance that inactivation of the gene that encodes *PTPN2* has been observed in 6% of T-ALL cases, and results in enhanced JAK1/STAT5 signaling upon IL-7 stimulation [134,135]. Interestingly, IL-7R signaling cascade can be hyperphosphorylated in patients who do not carry genetic aberrations in the *IL-7R*, *JAK*, or *STAT5* genes, suggesting that additional mechanisms exist to activate this pathway [136,137]. Coherently, recurrent loss-of-function mutations in the dynamin 2 (*DNM2*) gene were found to impair clathrin-dependent endocytosis of IL-7R leading to increased surface expression and enhanced IL-7R signaling in T-ALL cells. Thus, genetic evidence combined with transcriptional regulation of IL-7R by activated NOTCH signaling [93] and/or zinc finger E-box-binding homeobox 2 locus (ZEB2) overexpression [137], makes this signaling pathway highly attractive from a therapeutic stand point. Indeed, JAK inhibitors (ruxolitinib and tofacitinib) used in patients with rheumatoid arthritis [138] and myelofibrosis [139] could be repurposed for the treatment of T-ALL cases with documented activation of the IL-7R/JAK/STAT pathway. Thus, in the context of T-ALL pharmacological inhibition of JAK1/JAK2 by ruxolitinib has shown therapeutic activity in ETP-ALL primary patient xenografts in vivo in both the presence and the absence of JAK-STAT mutations [140]. Incidentally, the group headed by *Meijerink* found that mutations in the IL-7R signaling pathway (including *JAK1* and *KRAS* mutations) were associated with glucocorticoid resistance in patient samples, and that inhibition of Mitogen-activated protein kinase kinase/extracellular signal–regulated kinases (MEK/ERK) or PI3K/AKT/mTOR pathways enhanced steroid sensitivity in primary samples [141]. Surprisingly, the JAK1 inhibitor ruxolitinib had synergistic cytotoxic effects only in a minority of primary T-ALL samples, possibly due to the low proliferative capacity of primary T-ALL cells in vitro [141].

### 5.4. RAS Pathway

RAS proteins, including the isoforms Harvey rat sarcoma viral oncogene homolog (H-RAS), Neuroblastoma RAS viral oncogene homolog (N-RAS) and Kirsten rat sarcoma viral oncogene homolog (K-RAS), are small GTPases acting as molecular switches that oscillate between an inactive GDP-loaded and an active GTP-loaded conformation. RAS proteins transduce the signal from multiple cell surface receptors including the TCR, RTKs and cytokine receptors, to downstream effector pathways, such as the PI3K/AKT and MAPK pathways, to regulate a number of cell fate decisions. RAS genes are mutated in about one third of human cancers, with mutations in *N-RAS* being the most prevalent in hematological malignancies. Approximately 15% of all hematological tumors display point mutations in codons 12, 13 or 61 of *RAS* genes, impairing its GTPase function and leading to a constitutively active form of the protein [142]. Mutations in *N-RAS* and *K-RAS* are enriched in relapsed ALL patients and appear to be associated with steroid resistance [143,144]. Moreover, *K-RAS* mutations rendered lymphoblasts resistant toward methotrexate, while sensitizing them to vincristine [144]. Importantly, a conditional *K-Ras^G12D^* (where Glycine in codon 12 is mutated to Aspartic acid in *K-Ras*) knock-in mouse model develops T-cell leukemia/lymphoma [145] and *Ras* activating mutations cooperate with *NOTCH1* mutations to drive T-ALL development [146]. Moreover, RAS signaling is frequently hyperactivated in T-ALL patients [147], either by mutations in *RAS* itself [144,148,149,150] -especially in the early precursor subtype- [42] or by alterations in the activity or expression of other regulatory components of the pathway, mainly RAS GEFs (RAS guanidine nucleotide exchange factors) and RAS GAPs (RAS GTPase activating proteins). Indeed, neurofibromin 1 (*NF1*), a tumor suppressor protein enhancing RAS hydrolyzing activity, was reported to be mutated in T-ALL patients [151], as well as to have a leukemogenic potential in vivo when deleted in combination with p120-RAS GAP in T-cells [152]. Another peculiar mechanism of increased RAS signaling in T-ALL results from the overexpression of RAS guanine nucleotide-releasing protein 1 (RASGRP1), a RAS GEF normally highly expressed in T-cells and critical for thymocyte differentiation and signal transduction downstream of the TCR [153]. In T-ALL, RASGRP1 induces higher rates of GTP/GDP exchange and promotes cytokine-induced RAS downstream signaling, mainly via the PI3K/AKT axis [154,155]. Moreover, loss-of-function mutations in *PTPN11*, encoding the protein phosphatase non-receptor type 11, which negatively regulates the RAS pathway, have also been described [156].

### 5.5. ABL Kinase

*ABL1* gene encodes a ubiquitously expressed tyrosine kinase and it is found to be rearranged in 8% of T-ALL cases [157]. The resulting fused protein products display constitutive kinase activity and promote survival and proliferation pathways. While B-cell receptor (BCR)-ABL1 fusion protein following the Philadelphia translocation t (9; 22) (q34; q11.2) is a hallmark of chronic myeloid leukemia and is also common in precursor B-cell ALL (B-ALL) [158], BCR-ABL1-positive T-ALL patients are exceptionally rare [159]. The most frequent and T-ALL-specific ABL1 rearrangement is NUP214-ABL1 episomal amplification (6% cases), which was described in T-ALL cell lines, as well as in adult and pediatric patients associated with TLX1 or TLX3 expression and *CDKN2A* deletion [32,160,161]. Other less common partners of ABL1 that were sporadically reported in T-ALL also include ETV6 [162] and echinoderm microtubule-associated protein EML1 [163]. All the above-mentioned ABL1 fusion proteins render cells sensitive to small tyrosine kinase inhibitors such as imatinib mesylate (Glivec) [164,165]; however, the onset of resistance due to acquired mutations in *ABL1* gene has been observed [166].

### 5.6. NF-κB Pathway

Nuclear factor kappa B subunit (NF-κB) family of transcription factors includes five members, p50 (NF-κB1), p52 (NF-κB2), v-Rel avian reticuloendotheliosis viral oncogene homolog A (RelA or p65), v-Rel avian reticuloendotheliosis viral oncogene homolog B (RelB) and v-Rel avian reticuloendotheliosis viral oncogene homolog (c-Rel), which all contain a Rel homology domain (RHD) and form homo- and heterodimers with different transcriptional properties. In the resting state, inactive NF-κB dimers are retained in the cytoplasm by inhibitor of nuclear factor kappa B (IkB) proteins. Upon stimulation, IkB kinase (IKK) complex phosphorylates IkB targeting it for proteosomal degradation, thus allowing NF-κB dimers to translocate to nucleus and bind DNA. Depending on the initiating stimulus and the effector dimers that are activated, a canonical and a non-canonical pathway can be distinguished. NF-κB signaling exerts a pivotal regulatory role in innate and acquired immune responses and its alteration has been associated to inflammatory diseases and immune-deficiencies, as well as to solid and hematological tumors [167]. In T-cells, NF-κB signaling can be activated by a variety of immune signals, including antigens, Toll-like receptor ligands and inflammatory cytokines such as tumor necrosis factor-alpha (TNF-α) and interleukin 1β [168]. NF-κB was reported to be involved not only in early thymocyte development, promoting a pre-TCR-induced survival step between DN3 and DN4 stages [169], but also in antigen-dependent T-cell selection, lineage commitment and maturation. Indeed, different levels of NF-κB can regulate CD8^+^, but not CD4^+^, positive and negative selections [170]. Moreover, NF-κB signaling is also required for thymic T_reg_ development [171,172] and IFN-γ-producing NKT cell maturation [173,174]. Unlike other lymphoid malignancies, mutations in NF-κB signaling genes are rare in T-ALL, however constitutive activation of the pathway has been observed in T-ALL primary samples [175,176]. NOTCH1, which is mutated in half of T-ALL cases (see Section 5.1), can activate NF-κB pathway in T-ALL cell lines and in the intracellular NOTCH1 (ICN1)-induced mouse model, either transcriptionally, promoting RelB and p52 expression, or indirectly via IKK complex activation [177]. Moreover, NOTCH1 target HES1 was reported to repress cylindromatosis (CYLD), a deubiquitinase negatively regulating IKK complex, both in primary T-ALL samples and cell lines [178]. The critical role of this interaction is demonstrated by the anti-leukemic effects of NF-κB inhibition in T-ALL and the strict requirement of NF-κB signaling for NOTCH1-induced transformation. Besides NOTCH1, in vivo constitutive activation of NF-κB was also reported in ETV6-JAK2 [179], Tal1 [180] and NOTCH3 mouse models of T-ALL [181]. Other reports highlighted a pro-oncogenic role of NF-κB in T-ALL leukemogenesis by contributing to the crosstalk between leukemic T-cells and microenvironmental stromal cells [179,182]. Thus, NF-κB appears as an appealing target for T-ALL treatment. However, moderate results have been obtained in T-ALL, as demonstrated by modest increases in sensitivity to apoptosis of T-ALL cell lines treated with IKK inhibitors [177,178] or the proteasome inhibitor bortezomib [177]. The therapeutic value of NF-κB inhibition was also investigated in relapsed pediatric T-ALL patients, where treatment with bortezomib in combination with multiagent chemotherapy was shown to be more effective than single treatments [183].

### 5.7. Hedgehog Pathway

Hedgehog (HH) signaling is a conserved pathway essential for stem cell biology during embryonic development and tissue homeostasis and repair in adult life [184]. The canonical pathway can be activated by binding of different secreted ligands (Sonic Hedgehog, SHH; Desert Hedgehog, DHH; Indian Hedgehog, IHH) to transmembrane receptors Patched (PTCH1 or PTCH2). Patched is an unusual receptor because it is a tonic repressor of another transmembrane receptor, Smoothened (SMO). Thus, once Patched interacts with hedgehog ligands, SMO is activated. Activated SMO transduces the signal to Gli family of transcription factors (GLI1, GLI2, and GLI3), resulting in their nuclear translocation. GLI1 and GLI2 mostly act as transcriptional activators, while GLI3 as a repressor [184]. Besides a role, though controversial, in primitive and definitive hematopoiesis [185], HH also regulates early stages of thymic development, especially at the DN1-DN2 and DN-DP transition [186,187,188]. Indeed, immature T-cells moving across the thymus are exposed to different concentrations of stroma-secreted HH ligands, resulting in variable levels of pathway activation and specific patterns of expression of GLI genes and their targets [187,189,190].

HH signaling is aberrantly activated by oncogenic mutations or autocrine/paracrine/inverse paracrine mechanisms in a variety of solid and hematological malignancies, including acute and chronic myeloid leukemia and multiple myeloma, contributing to tumor development and expansion [191,192], as well as cancer stem cell maintenance [193,194,195]. In the context of T-ALL, evidence of the involvement of Hedgehog signaling is emerging. Recent studies showed that pathway components are expressed and signaling is active in some T-ALL cell lines and primary samples [196,197]. In addition, rare somatic mutations in HH pathway members were found in T-ALL, including two truncating mutations in *SMO* (R726 * and R763 *) and missense mutations in *GLI1* (S538F) and *GLI3* (G727R) [198]. Additionally, the same group reported that the HH cascade was active in ≈20% of the patient samples, through ectopic expression of SHH and IHH and of the downstream GLI1 transcription factor, suggesting that the crosstalk with other pathways such as IL-7, vascular endothelial growth factor (VEGF) or NOTCH signaling pathways and the overall transcriptional deregulation intrinsic to T-ALL, rather than pathway-intrinsic mutational events, are responsible for this activation [197]. Moreover, ectopic expression of HH ligand in *JAK3* (M511L) mutant mouse model of T-ALL induces a growth advantage, higher infiltration rates, and thymic epithelial cell activation indicating a supportive role in leukemia development. However, Gao et al. found HH signaling to be dispensable for NOTCH-induced leukemia induction and progression [196]. The debated effects of Smo deficiency in normal hematopoiesis [199] and leukemogenesis [196], together with the reduced efficacy of SMO inhibitors (cyclopamine and GDC-0449) on cell line growth with respect to GLI1 inhibitors [197,200], underscores the importance of non-canonical Smo-independent modulation of HH signaling and the necessity of dissecting the complex regulatory network upstream of GLI1 [201]. Indeed, amounting evidence indicate that ligand-independent hedgehog signaling, also named non-canonical hedgehog signaling, not sensitive to SMO inhibitors plays an essential role in cancer [202]. Thus, given the modest activity of the FDA-approved SMO inhibitors against xenografts of human T-ALL and the known issue of emergence of resistance to these drugs in other cancer clinical trials, it will be imperative to determine which signaling pathways alter sensitivity to HH pathway inhibitors. These findings will be crucial to design rational combination therapies incorporating HH pathway inhibitors in T-ALL.

### 5.8. Calcineurin/NFAT Pathway

Calcineurin (Cn) is a calcium-dependent serine/threonine phosphatase composed of a catalytic subunit (PPP3CA or CnA) and a regulatory subunit (PPP3CB or CnB), implicated in a variety of physiological and developmental processes in nervous, cardiovascular, musculoskeletal and immune system [203]. Upon surface receptor stimulation, increased levels of intracellular calcium are detected by sensor protein calmodulin, which in turn enhances calcium-induced Cn activation. Calcineurin dephosphorylates a number of substrates, prominently NFAT family members, allowing their nuclear translocation and transcriptional activity. In normal T-cells, the cooperation between NFAT factors with different transcriptional partners leads to the expression of distinct set of genes, including inflammatory cytokines, thus eliciting multiple effects [204]. Cn/NFAT pathway is known to induce cytokine gene expression in activated T-cells upon TCR engagement and its essential function is underscored by the use of calcineurin inhibitors such as cyclosporine (CsA) and FK506 as immunesuppressor drugs in transplantation medicine [79]. A role for NFAT members has also been described in positive selection during thymocyte development, as demonstrated by CnAβ^−/−^ and CnB^−/−^ conditional knock-out models [205,206], in T helper cell (T_H_) T_H1_ versus T_H2_ differentiation [207] and in the induction of anergy [208].

Thus, the Cn/NFAT signaling pathway is implicated in numerous biologically relevant processes, connecting perturbations in calcium signaling to gene expression. Recently, this signaling pathway has been implicated in the induction and progression of hematological malignancies [209]. In fact, nuclear NFAT2 was found in cases of Burkitt’s lymphoma, diffuse large B cell lymphoma and aggressive T-cell lymphoma [210,211]. Surprisingly, in fibroblasts, distinct and opposing roles for the transcription factors NFAT1 and NFAT2 in tumorigenesis were revealed in which NFAT1 functions as a tumor suppressor and NFAT2 as an oncogene [212]. In T-ALL, Cn was reported to contribute to leukemogenesis in ICN1 and ETV6-JAK2 mouse models of T-ALL, in which sustained activation of the pathway by microenvironmental cues leads to constitutive desphosphorylation of NFAT. Inhibition of Cn by CsA and FK506 could induce significant anti-leukemic effects leading to rapid disease remission and improved survival. On the other hand, ectopic expression of a constitutively active Cn mutant accelerated leukemia progression and invasiveness [213]. The molecular mechanisms that account for sustained activation of Cn in leukemic cells remain to be identified, but may require signaling from the *in vivo* tumor microenvironment and have been shown to be independent of TCR and pre-TCR expression (the main Cn activators in normal T progenitors), at least in the ETV6-JAK2 mouse model [213]. Recent developments have further strengthened the role of Cn activation in the pathogenesis of T-ALL. Indeed, elegant work demonstrated that conditional deletion of *CnB1* in leukemic cells impairs leukemia propagation, reduced survival and homing upsetting adhesive interactions between leukemic cells and their supportive stroma [214]. In addition, Cn activation was found to be critical for leukemia initiating/propagating cell activity as Cn-deficient leukemic cells were unable to transplant the disease to syngeneic recipient mice [214]. A limitation of the therapeutic pre-clinical findings is that currently available Cn inhibitors appear suboptimal as therapeutic agents, since they are associated with serious side effects [215], show off-target effects in T-ALL cells [214], and potentially interfere with the anti-tumor immune response. Thus, it is not surprising that alternative options have been explored to dampen Cn signaling. In fact, several groups have tried to identify and target molecular pathways acting downstream of Cn and critical in T-ALL biology. Stemming from this consideration, recent work identified a series of Cn-dependent genes in T-ALL including cell cycle inhibitors (notoriously difficult to target therapeutically), and genes implicated in adhesion/migration [214]. The authors followed-up on these findings and found that Cn regulates the adhesive/migratory properties of T-ALL cells by increasing C-X-C motif chemokine receptor 4 (CXCR4) surface expression in a cortactin-dependent way [216]. The ligand for this chemokine receptor, C-X-C motif chemokine ligand 12 (CXCL12) or stromal-derived growth factor-1 (SDF-1) is of particular importance as it is secreted by numerous cell types in the bone marrow such as osteoblasts lining the bone endosteum and endothelial cells [217] and is implicated in the homing of hematopoietic stem cells to the bone marrow [218]. Recent studies have demonstrated that the critical source of CXCL12 is the endothelial compartment of the vascular niche [219]. Inactivation of CXCL12 in the vascular niche or CXCR4 in T-ALL cells impairs their LIC activity in both murine T-ALL and human xenografts [216,219]. Of note, another CXCL12 binding receptor has been identified and called C-X-C motif chemokine 7 (CXCR7) [220,221], whose relevance to T-ALL biology is still ill-defined. In fact, T-ALL cells express CXCR7 and it may potentiate CXCR4 responses to CXCL12 [222]. Recently, we undertook a more proteomic approach to identify putative downstream targets/effectors contributing to the pro-oncogenic activity of Cn in T-ALL. Indeed, we used tandem affinity chromatography followed by mass spectrometry to identify Cn-interacting proteins in T-ALL cells. We found that the isolated proteins were implicated in numerous key signaling pathways, including eukaryotic initiation factor 2 (eIF2) signaling, cell cycle control, mTOR signaling and 14-3-3 mediated signaling [223]. Systematic inhibition of the top signaling pathways enriched in our CnA protein complex identified a highly synergistic drug interaction between inhibition of Cn and the PI3K/AKT/mTOR signaling pathway. Further studies showed that AKT represented the critical node of the pathway to inhibit to obtain the most synergistic cytotoxic effect with Cn inhibitors. This cytotoxic effect was prevalently due to the strong reduction in the expression of the anti-apoptotic protein myeloid leukemia 1 (MCL-1). Our studies also uncovered a complex interaction between GSK-3β and Cn/NFAT signaling in T-ALL cells [224]. In fact, in resting cells Glycogen Synthase Kinase 3 (GSK-3) participates in maintaining NFAT proteins in their inactive hyper-phosphorylated form, antagonizing Cn/NFAT signaling [225,226]. GSK3 is a constitutively active kinase that is phosphorylated and inactived by PI3K and AKT signaling [227]. Surprisingly, we found that CnA was able to directly interact with GSK-3β and increase its kinase activity possibly by augmenting its autophosphorylation. These results reiterate the complex role of GSK-3 in cancer, as this kinase has been shown to act as a tumor suppressor in certain tumors, whereas in others it acts a tumor promoter [228]. We found that GSK-3β acts mainly as a tumor promoter in T-ALL by promoting the stabilization of proteins such as MCL-1, c-MYB, and possibly X-linked inhibitor of apoptosis (XIAP). Interestingly, dual Cn and GSK-3β inhibition showed a synergistic anti-leukemic effect in vitro and in vivo, via downregulation of anti-apoptotic proteins such as XIAP and claspin [224]. In conclusion, all these studies highlight how the Cn/NFAT pathway links microenvironmental derived signals with the intrinsically altered signaling pathways found in T-ALL and provide additional targets and perspectives for future therapeutic strategies.

### 5.9. Wnt Signaling

Wnt signaling plays an important role in normal hematopoiesis [229,230], and it often becomes de-regulated in malignancies of the hematological system [231]. Briefly, the Wnt signaling cascade is often discerned into canonical or Wnt/β-catenin pathways and the non-canonical pathways. In the canonical Wnt pathway, in the absence of Wnt ligands, cytoplasmic levels of β-catenin are kept very low due to its constitutively phosphorylation and degradation through the action of a protein complex. This so called destruction complex is composed of two negative regulatory kinases, GSK3β and casein kinase 1 (CK1), and at least two anchor proteins, Axin1 or Axin2 and adenomatous polyposis coli (APC) protein. APC and Axin sequester β-catenin in the cytoplasm. Upon binding of Wnt ligands to the Frizzled receptor and LDL receptor related protein 6 (LRP6), the destruction complex is inactivated, allowing the accumulation of dephosphorylated β-catenin and its migration to the nucleus where it binds members of the T-cell factor (TCF)/Lymphoid enhancer binding factor (LEF) transcription factor family and activates the transcription of Wnt-responsive genes [232]. It is very likely that both the microenvironment (thymus) and the cell of origin of leukemia (immature T-cell) will greatly influence how normal Wnt signaling in these cells is deregulated and may subsequently lead to malignant transformation [233]. Thus, the interplay between microenvironmental signals, such as Wnt ligands and antagonists in combination with the expression of intracellular components, such as TCF/LEF transcription factors and intracellular inhibitors (such as β-catenin interacting protein, ICAT), in the progenitor cells themselves will influence how the normally tightly regulated Wnt signaling levels may predispose to malignant transformation. Signaling pathways and oncogenes aberrantly expressed may also influence Wnt signaling, helping to tip the balance. Deregulation of Wnt signaling is a frequent event in cancer, however its role in the pathogenesis of T-ALL has only recently been unveiled. In the study by *Guo* et al. [234], a constitutively active form of β-catenin was used that was expressed from the DN3 stage onwards and determined the accumulation of DP thymocytes which were predisposed to malignant transformation. The resulting leukemias were associated with c-MYC upregulation but did not develop *NOTCH1* mutations. These findings, together with the report that human T-ALL cases without *NOTCH1* mutations show high Wnt signaling levels [235], suggest that aberrant Wnt signaling may be a leukemia initiating event similar to NOTCH signaling. On the other hand, two recent studies show an important tumor suppressor role for T-cell factor 1 (TCF1) in T-ALL development [236,237]. In both studies, mice deficient for Tcf1 are highly susceptible to develop phenotypically heterogeneous leukemias (due to several incomplete successive T-cell developmental blocks). In fact, it was found that upon *Tcf1* deletion, LEF1 protein levels become deregulated in all thymic subsets, resulting in abnormally high levels of the long isoform of LEF1, thus predisposing thymocytes to leukemic transformation. Interestingly, a key role for LEF1 has been found in mouse models of leukemias induced by activated forms of NOTCH [238]. In fact in these lymphomas, NOTCH1 was found to directly transcriptionally regulate LEF1 expression. Hence, deregulation of LEF1 expression, either via lack of the tumor suppressor TCF1 or aberrant activation of NOTCH pathway accelerates lymphomagenesis. These findings in the mouse appear relevant for human T-ALL. In fact deletions and mutations in *LEF1* that inactivate its expression has been found in T-ALL [239], and more recently loss of TCF1 has been reported in a subset of pediatric T-ALL, the ETP subgroup, in which two patients were found to have deletions of Transcription factor 7 (*TCF7*) [the gene encoding TCF1] [237]. Additionally, gene expression profiling of *Tcf7^−/−^* lymphomas showed upregulated expression of myocyte enhancer factor 2c (Mef2c) [236], which has been recently associated with an immature T-ALL subgroup [26]. In conclusion, these results indicate that TCF1 may act as a tumor suppressor in certain subgroups of pediatric T-ALL (ETP-ALL and MEF2C positive T-ALL). Recently, a further twist to the role of Wnt signaling has been added. In fact, active Wnt signaling has been shown to be restricted to minor subpopulations within bulk tumors, and these Wnt-active subsets were highly enriched for leukemia initiating activity (i.e., putative T-ALL stem cells) [240]. In these stem cells, hypoxia-inducible factor α (HIF-1α) was also activated. Genetic inactivation of β-catenin or HIF-1α severely reduced stem cell frequency, while having minimal impact on the growth or viability of bulk tumor cells, implying that elements of Wnt and Hif pathways specifically support leukemic stem cells.

### 5.10. Altered Metabolic Homeostasis

Metabolic homeostasis is a fundamental trait of cells which becomes altered in cancer in order to satisfy the heightened demand for metabolites necessary for growth and proliferation. Oncogenic mutations can directly alter cellular metabolism in a cell-intrinsic way, favoring malignant transformation. Moreover, microenvironmental cues such as hypoxia, nutrient availability, oxidative stress and cross-talk from neighboring cells all affect cancer cell metabolism and contribute to determine metabolic heterogeneity within the tumor [241]. It is thus becoming clear that the metabolic phenotype of cells within tumors is heterogeneous (some cells within the tumor are predominantly glycolytic, whereas others have primarily a oxidative phosphorylation (OXPHOS) metabolic phenotype) also due to the metabolic milieu of the tumor microenvironment. Leukemia is no exception as these cells often expand in hypoxic areas such as the bone marrow or the thymus [242,243]. Moreover, in T-ALL many of the metabolic regulators that allow metabolic adaptation such as PI3K/AKT/mTOR, NOTCH1, c-MYC, AMPK, and HIF-1 are frequently deregulated, leading these cells to exhibit increased glucose consumption and increased glycolysis [244]. T-ALL cells are also highly dependent on glutamine, as this metabolite can be used to produce glutathione, amino acids, hexosamine (amino sugars involved in the synthesis of glycosylated molecules), nucleotides, and fuel mitochondrial Krebs cycle through the production of α-ketoglutarate thus allowing the synthesis of pyruvate, NADPH, acetyl-CoA, or citrate [241]. A key regulator of glutamine metabolism (glutaminolysis) is the transcription factor MYC. Indeed, this oncogenic transcription factor regulates factors implicated in glutaminolysis and glutamine uptake such as glutamine transporters (MCT1) and glutaminase [245]. MYC is often de-regulated in T-ALL (see Section 5.1) as a consequence of its transcriptional activation by NOTCH1, and coherently supports NOTCH1 induced transformation. Recently, using a NOTCH1-dependent mouse model of T-ALL, inhibition of NOTCH1 signaling through the use of GSI attenuated glutaminolysis, rendering cells dependent on autophagy to support metabolism and survival [58]. Moreover, glutaminase inhibition synergized with NOTCH1 inhibition to inhibit leukemia growth. Significantly, *PTEN* deletion, rendered T-ALL cells resistant to gamma secretase inhibition by upregulating glycolysis [58].

Recent evidence suggests that AMPK confers cancer cells with the ability to cope with metabolic stresses by regulating glucose metabolism. AMPK is a heterotrimeric serine/threonine kinase composed of an α-catalytic subunit and two regulatory subunits β and γ which regulate its activation and substrate specificity [246]. AMPK is a cellular fuel sensor, activated under conditions of ATP depletion and elevated AMP levels such as nutrient depletion, hypoxia and other metabolic or environmental stresses [247,248]. Activation of AMPK by falling energy status, promotes metabolic homeostasis by activating ATP-generating pathways such as glucose uptake, glycolysis, fatty acid uptake and oxidation, and mitochondrial biogenesis on the one hand and by inactivating ATP-consuming processes such as fatty acid, cholesterol and protein synthesis. In cancer cells, activation of AMPK can also suppress proliferation and growth through multiple mechanisms, including p53 activation, inhibition of mTORC1 pathway, or activating transcriptional responses [247]. Thus, the role of AMPK in cancer is not yet fully understood, as it seems to have both tumor suppressive and promoting roles, depending on the cellular context. AMPK can thus have growth suppressive functions in certain cancer settings [249] such as the Eμ-myc induced lymphoma model, in which deletion of the α1 catalytic subunit of AMPK promoted lymphomagenesis. Further, pharmacological activation of AMPK can slow the growth of some tumors such as breast cancer [250]. In hematological malignancies such as T-ALL, AMPK has been shown to restrain tumor growth [94], and AMPK activation (mainly using agonists like metformin or AICAR) suppresses leukemia cell growth through inhibition of mTORC1 [251], p38 mitogen activated protein kinase (p38 MAPK) [252] or unfolded protein response (UPR) signaling [253]. Conversely, multiple oncogenic signals, including RAS and MYC, can generate metabolic stress [254,255] and AMPK may promote cancer cell survival under these conditions. Indeed, AMPK may be important to mitigate metabolic stress in myeloid leukemia initiating cells (LICs) [256] and activated T-cells in vivo [257]. In fact, in mouse models of acute myeloid leukemia and chronic myeloid leukemia [256], deletion of the α1 catalytic subunit of AMPK depleted LICs and prolonged leukemia free survival through reduced glucose uptake and glycolysis, determining depletion of reducing agents such as NADPH and glutathione. This leads to increased oxidative stress and DNA damage accumulation in LICs. Moreover, AMPK inhibition was found to enhance apoptosis in MLL-rearranged pediatric B-ALL cells [258]. An elegant study recently compared the metabolic programs of primary T-ALL and normal activated T-cells [259]. Surprisingly, this study found that although T-ALL cells utilize aerobic glycolysis this usage is restrained compared to normal proliferating T-cells [259]. Moreover, NOTCH1 overexpression induced metabolic stress that led to AMPK activation that acted to restrain glycolysis through inhibition of mTORC1 and promoted mitochondrial oxidative metabolism and mitochondrial Complex I activity to mitigate stress. Thus, exacerbating this stress by AMPK or mitochondrial inhibition may provide a novel therapeutic approach for T-ALL.

## 6. Conclusions

T-ALL is an aggressive hematological disease for which few therapeutic options are available in the case of primary resistant or relapsed disease, highlighting the need for better risk stratification and the necessity to identify more effective targeted therapies [4]. Although, in the past decades, huge progress has been made in our understanding of the genetic landscape and molecular pathogenesis behind T-ALL, some gaps in our knowledge are present. In fact, dissecting the intricate signaling networks governing the interaction between environmental permissive cues and oncogenic cell-intrinsic factors leading to metabolic homeostasis of leukemia cells will contribute to fully capture critical steps of T-ALL pathogenesis. Thus, the wealth of information gained in the past decades on the role of oncogenes and tumor suppressors, together with cutting-edge studies evaluating the genetic and epigenetic landscape of T-ALL will need to be combined with a better understanding of the leukemic “niche” to design effective targeted therapies for the treatment of T-ALL.
